# Effects of Polyether Amine Canopy Structure on Heavy Metal Ions Adsorption of Magnetic Solvent-Free Nanofluids

**DOI:** 10.3390/nano14060505

**Published:** 2024-03-11

**Authors:** Qi Zhang, Jian Zhang, Jian Shi, Ruilu Yang

**Affiliations:** 1 Analysis and Testing Center, Nantong University, Nantong 226019, China; 2118310010@stmail.ntu.edu.cn (Q.Z.); shi.j1@ntu.edu.cn (J.S.); 2 School of Transportation and Civil Engineering, Nantong University, Nantong 226019, China

**Keywords:** magnetic Fe_3_O_4_, solvent-free nanofluids, heavy metal adsorption

## Abstract

Three Fe_3_O_4_ magnetic solvent-free nanofluids with different amine-based coronal layer structures are synthesized and characterized by using magnetic Fe_3_O_4_ as the core, silane coupling agent as the corona, and polyether amines with different graft densities and chain lengths as the canopy. The concentration of heavy metal ions after adsorption is measured by atomic absorption spectrometry (AAS) to study the effect of Fe_3_O_4_ magnetic solvent-free nanofluids on the adsorption performance of the heavy metal ions lead (Pb(II)) and copper (Cu(II)) in water. The adsorption of Fe_3_O_4_ magnetic solvent-free nanofluid was explored by changing external condition factors such as adsorption contact time and pH. Additionally, the adsorption model is established. The magnetic solvent-free nanofluid is separated from water by applying an external magnetic field to the system, and desorption and cyclic adsorption tests are carried out. Based on the adsorption mechanism, the structure design of Fe_3_O_4_ magnetic solvent-free nanofluid was optimized to achieve optimal adsorption performance.

## 1. Introduction

With the accelerated development of industrialization, significant quantities of wastewater carrying heavy metal ions are released into the environment [1]. These heavy metals such as copper and lead ions are highly soluble in water, non-biodegradable, and easily absorbed by organisms, posing a serious threat to human health and ecological security once they enter the biological chain [2,3]. Current methods for the removal of copper and lead ions in wastewater include, but are not limited to, flocculation, redox reactions, ion exchange, and adsorption [4,5,6]. Among these methods, adsorption is preferred owing to its high removal efficiency, cost-effectiveness, ease of operation, and widespread application [7,8,9]. However, traditional adsorption materials have limitations such as narrow pH range, relatively poor selectivity, and low adsorption capacity, which do not fully meet practical needs [10,11]. Therefore, there is an urgent need to explore new heavy metal ion adsorption materials with high adsorption capacity, rapid adsorption and desorption capabilities, and easy separation and regeneration.

Magnetic nanoparticles have the advantage of strong adsorption capacity, large specific surface area, and ease of separation under an external magnetic field, making them promising for the adsorption of heavy metal ions in water [12,13]. However, magnetic nanoparticles tend to agglomerate due to their high surface energy, which reduces the contact surface area between nanoparticles and heavy metal ions, thus limiting their adsorption capacity. Additionally, the lack of surface active adsorption sites makes direct application difficult. Organic/inorganic nanocomposite systems, which combine the properties of inorganic nanoparticles and organic matter with customizable structure and performance, hold potential for the adsorption of heavy metal ions in water. By grafting oligomers onto the surface of the modified inorganic nanoparticles through ionic or covalent bonds, solvent-free nanofluids with fluidity that are capable of solid–liquid transition without solvent and at room temperature can be prepared [14,15,16,17,18]. Due to the customizable structure, when amine oligomers capable of complexing with heavy metal ions in water are grafted onto the surface of magnetic nanoparticles of Fe_3_O_4_, magnetic solvent-free nanofluids are formed. These combine the advantages of strong adsorption capacity, large specific surface area, and easy separation of magnetic nanoparticles with the strong ability of amine groups to capture heavy metal ions. This type of magnetic solvent-free nanofluid can be applied as a material for the adsorption of heavy metal ions. Furthermore, the entire system has zero vapor pressure and excellent thermal stability, and remains in a fluid state under solvent-free and room-temperature conditions, addressing the issue of magnetic nanoparticle agglomeration. However, nanoparticles can also be toxic [19]. Polyetheramine has low toxicity, and its environmental impact is less significant compared to heavy metal ions.

Regarding the synthesis of magnetic solvent-free nanofluids, Mehrali et al. prepared a solvent-free nanofluid based on Fe_3_O_4_/GO, a superparamagnetic fluid material within the environmental context [20]. Additionally, Zheng et al. synthesized solvent-free nanofluids using Fe_3_O_4_/MWCNTs as multicomponent nanocores [21]. Furthermore, Bai et al. introduced a magnetic solvent-free nanofluid based on Fe_3_O_4_/polyaniline (PANI) for the first time, showcasing broad application potential, including magnetic field detection [22]. Moreover, there has been research on the adsorption of heavy metals in water using amine graft-modified magnetic nanoparticles. Tan et al. developed amino-functionalized magnetic particles to adsorb lead ions in water, obtaining a maximized adsorption capacity of 40.10 mg·g^−1^ [23]. Xin et al. synthesized amino-functionalized Fe_3_O_4_ particles for the adsorption of lead, cadmium, and copper ions, demonstrating a high removal rate of metal ions in aqueous solutions, reaching 98% [24]. Additionally, Tan et al. successfully prepared EDTA-modified nanomaterials based on chitosan-modified Fe_3_O_4_, exhibiting an adsorption capacity of 210 mg·g^−1^ for lead ions [25]. Mahdav et al. successfully synthesized a silicon dioxide (SiO_2_)-coated Fe_3_O_4_-modified amine material, which can effectively remove lead ions [26]. Based on the above research, it is evident that amine-modified magnetic nanoparticles acting as heavy metal ion adsorption materials are mainly solid materials, with limited reports on magnetic solvent-free nanofluids in this capacity. Yang et al. prepared a novel magnetic loading porous liquid absorbent, displaying outstanding adsorption performance, and the adsorption of Pb(II) and Cu(II) was 357.14 and 322.58 mg·g^−1^, respectively [27]. Furthermore, while numerous studies have focused on adsorption properties, there is a scarcity of research on the effect of the structure of adsorbed materials on the adsorption properties of metal ions. Therefore, it is crucial to delve into two pertinent factors related to the adsorption performance of magnetic solvent-free nanofluids on heavy metal ions: the impact of the graft density of the amine-based canopy and the influence of the length of molecular chains of the canopy.

Therefore, the purpose of this work is to obtain different types of Fe_3_O_4_ magnetic solvent-free nanofluids by adjusting the length of molecular chains and graft density of the amine-based canopy. The study aims to investigate the relationship between the adsorption behavior of heavy metal ions and the structure of Fe_3_O_4_ magnetic solvent-free nanofluids, and reveal the mechanism of Fe_3_O_4_ magnetic solvent-free nanofluids to capture heavy metal ions in water. This research aims to lay the theoretical foundation for the synthesis of new high-performance heavy metal ion adsorption materials.

## 2. Materials and Methods

### 2.1. Materials

Polyetheramine M2070 (MW~2000) and M1000 (MW~1000) were employed as the canopies, which were from Dalian Lianhao Inc. (Dalian, China) NH_3_·H_2_O (25~28 wt.%, AR), and Trimethoxysilane (KH560, 98 wt.%) were provided by Chengdu Aikeda Chemicals Co., Ltd. (Chengdu, China). FeCl_2_·4H_2_O (≥99.0%, GR), FeCl_3_·6H_2_O (≥99.0%, AR), and C_2_H_5_OH (≥99.7%, AR) were from Aladdin Technology Co., Ltd. (Shanghai, China). Dialysis bags (3500 Da) were provided by Solarbio Technology Co., Ltd. (Beijing, China) HCl (36–38%, AR) and CH_3_OH (≥99.5%, AR) were produced by Fuchen Chemical Co., Ltd. (Xi’an, China). Milli-Q Direct 8 was used to produced ultrapure water, which was purchased from Lightsun Technology Co., Ltd. (Shenzhen, China).

### 2.2. Preparation of Magnetic Solvent-Free Nanofluid

Fifty milliliters of ultrapure water was used to dissolve 0.0004 mol FeCl_3_·6H_2_O and 0.0002 mol FeCl_2_·4H_2_O, respectively. The solutions were subjected to 10 min of sonication and then mixed. After 30 min of sonication with stirring, 0.26 g ammonia and 0.39 g ultrapure water were added. Subsequently, the reaction was maintained at 35 °C for 30 min. This was followed by washing the product with ultrapure water about 7 times. The product was dried at 60 °C and then ground. Fe_3_O_4_ was obtained.

First, 30 g and 5 g methanol were used to dissolve 10 g polyetheramine-M2070 and 1.16 g KH560, respectively. The methanol and KH560 mixed solution was then added drop by drop to the methanol and M2070 mixed solution, stirring during the addition. Subsequently, 2 mL ultrapure water was added to the above solution and then reacted at 45 °C for 12 h with stirring. Separately, 0.5 g Fe_3_O_4_ was added to 30 g methanol and subjected to ultrasonication for 30 min. The resulting solution was then added to the reaction system and allowed to react for 12 h at room temperature. Ultrapure water was then used to dialyze the mixture for 48 h, with 4 changes of water during this period. Finally, following dialysis, Fe_3_O_4_(0.5 g)/KH560/M2070 was obtained after drying at 70 °C.

Fe_3_O_4_(0.25 g)/KH560/M2070 and Fe_3_O_4_/KH560/M1000 were also prepared following the same procedure. In the synthesis process, M2070 was 10 g and M1000 was 5 g.

### 2.3. Characterization

The micromorphology and structure of the adsorption materials were investigated through the scanning electron microscope (SEM, ZEISS G300, ZEISS, Jena, Germany) and the transmission electron microscope (TEM, Talos F200x, Thermo Fisher Scientific, Waltham, MA, USA). Elemental analysis was conducted using an energy dispersive spectrometer (EDS, Super X G2, Thermo Fisher Scientific, MA, USA). The structural properties were examined using the Fourier Transform Infrared spectrometer (FTIR, Tensor 27, Bruker, Ettlingen, Germany) in 400–4000 cm^−1^ spectrum range at room temperature. The STA 449 F5 device (NETZSCH, Welmar, Germany) was applied to perform thermogravimetric analyses (TGA, NETZSCH, Germany) under N_2_ protection, with a temperature rise rate of 10 °C·min^−1^. X-ray photoelectron spectroscopy (XPS, Axis Ultra DLD, Kratos, Kawasaki, Japan) was used to analyze group characteristics.

### 2.4. Adsorption Equilibrium and Kinetics

The adsorption equilibrium experiments for Cu(II) and Pb(II) were conducted using a set of conical flasks whose speed of stirring was set at 200 rpm. The adsorptions of Cu(II) or Pb(II) were measured in separate conical flares. The concentrations of Cu(II) and Pb(II) were analyzed using an atomic absorption spectrometer (AAS, tas-990F). Each set of experiments was repeated three times and the results were averaged. The amount of Cu(II) and Pb(II) adsorbed by adsorbents was calculated using Formulas (1) and (2), respectively:

(1) $$Q_{e}=\frac{(C_{0}-C_{e})V}{m}$$

(2) $$Q_{t}=\frac{(C_{0}-C_{t})V}{m}$$

where *Q_e_* (mg·g^−1^) and *Q_t_* (mg·g^−1^) were the adsorption capacities at equilibrium and time t, respectively. *C_e_* (mg·L^−1^), *C*_0_ (mg·L^−1^) and *C_t_* (mg·L^−1^) represented the initial, equilibrium, and time t concentrations of Cu(II) and Pb(II). m denoted the mass of the adsorbents up to 4 mg. V represented the volume of the solution, which was 100 mL.

### 2.5. Recyclability Test

For the purpose of cyclic utilization, adsorbents were introduced into 1 mol·L^−1^ HCl solutions and subjected to ultrasonic treatment for 30 min to promote the desorption of heavy metal ions. Subsequently, the separation of adsorbents from the HCl solution was achieved using an external magnetic field. The materials were then thoroughly washed with ultrapure water. Following this, the adsorbents were dried at 70 °C for 24 h to prepare them for reuse. To evaluate the adsorbents’ reusability, the adsorption–desorption experiments were repeated 5 times.

## 3. Results and Discussion

**Figure 1:**
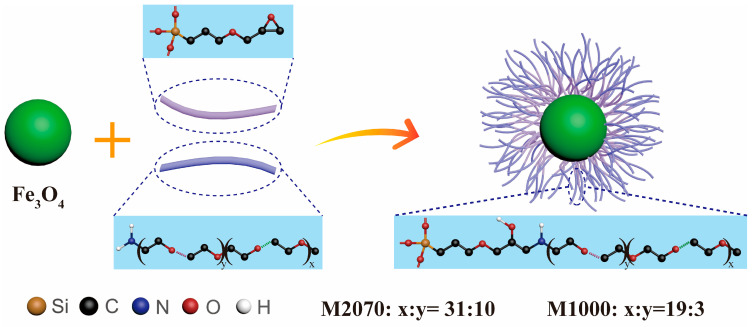
The synthetic process of magnetic solvent-free nanofluids (methyl and methylene hydrogen atoms are not shown for clarity).

Three magnetic solvent-free nanofluids were successfully synthesized using Fe_3_O_4_ as the core, (3-glycidoxypropyl) trimethoxysilane (KH560) as the corona, and different polyetheramines (M2070, M1000) as the canopy. The difference between Fe_3_O_4_(0.5 g)/KH560/M2070 and Fe_3_O_4_(0.25 g)/KH560/M2070 lies in the grafting densities. The distinction between Fe_3_O_4_/KH560/M2070 and Fe_3_O_4_/KH560/M1000 lies in the molecular chain length. The synthetic process of magnetic solvent-free nanofluids is shown in Figure 1.

**Figure 2:**
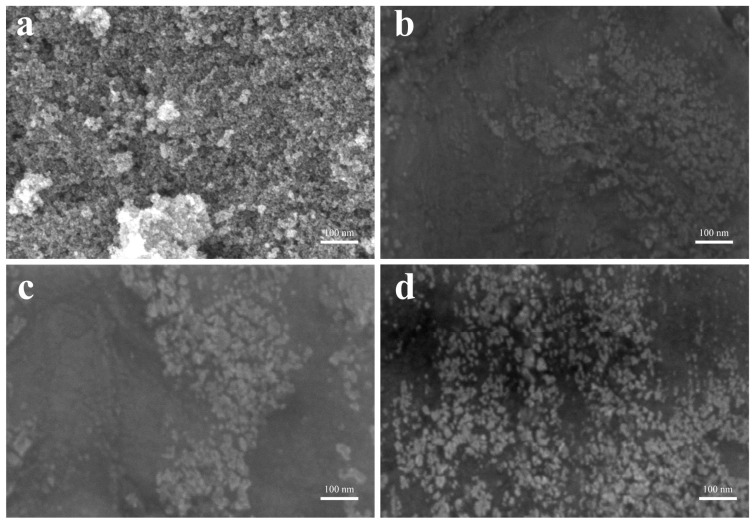
SEM images of (**a**) Fe_3_O_4_ powder, (**b**) Fe_3_O_4_(0.5 g)/KH560/M2070, (**c**) Fe_3_O_4_(0.25 g)/KH560/M2070, (**d**) Fe_3_O_4_/KH560/M1000.

The three magnetic solvent-free nanofluids were tested by SEM, FTIR, and TGA. The characterization results are as follows:

Figure 2 shows the SEM images of Fe_3_O_4_ nanoparticles and nanofluids. The black dots in the image of Figure 2a are Fe_3_O_4_ nanoparticles. It can be seen that Fe_3_O_4_ powder nanoparticles presented an obvious agglomeration phenomenon and stacked on top of each other, resulting in high surface energy. However, after grafting the organic oligomeric chains, no agglomeration was observed in the nanofluids samples shown in Figure 2b–d. This good dispersion was due to the organic layers coated on the Fe_3_O_4_ nanoparticles, and the organic coatings played an important role in protecting the nanoparticles from aggregation.

**Figure 3:**
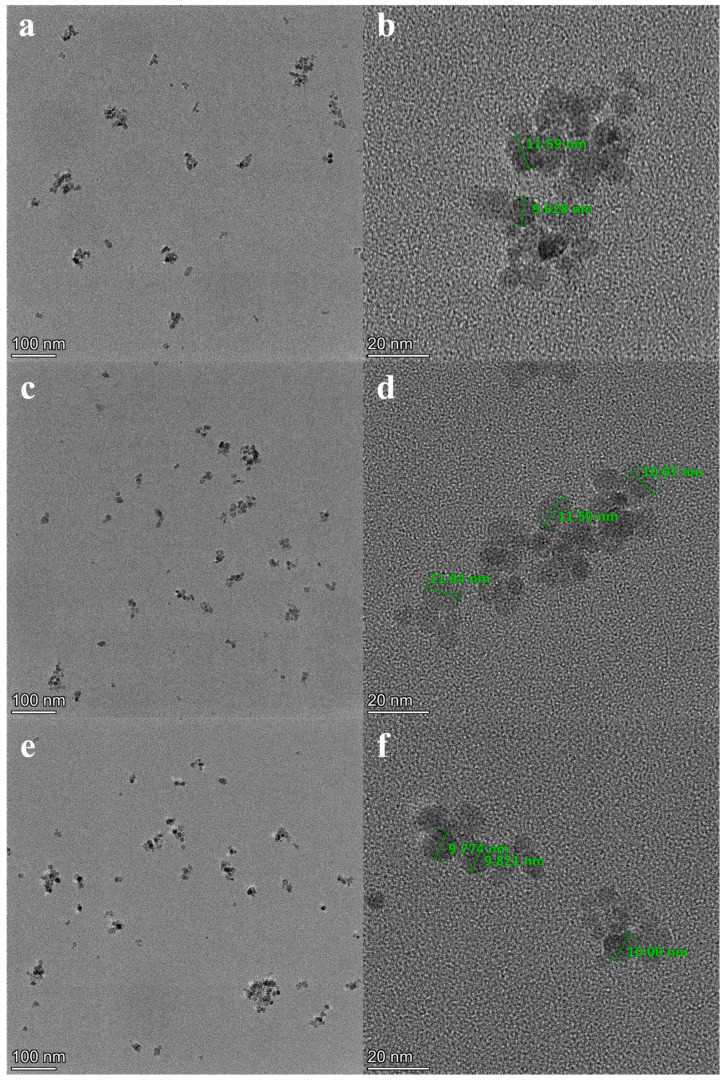
TEM images of (**a**,**b**) Fe_3_O_4_(0.5 g)/KH560/M2070, (**c**,**d**) Fe_3_O_4_(0.25 g)/KH560/M2070, (**e**,**f**) Fe_3_O_4_/KH560/M1000.

Illustrated in Figure 3 are TEM images of Fe_3_O_4_(0.5 g)/KH560/M2070, Fe_3_O_4_(0.25 g)/KH560/M2070, and Fe_3_O_4_/KH560/M1000. In Figure 3, it can be clearly observed that the size of Fe_3_O_4_ nanoparticles in all three materials was about 10 nm.

**Figure 4:**
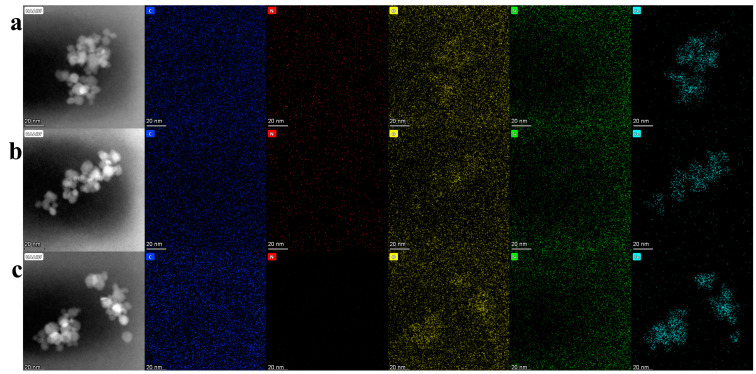
EDS maps of (**a**) Fe_3_O_4_(0.5 g)/KH560/M2070, (**b**) Fe_3_O_4_(0.25 g)/KH560/M2070, (**c**) Fe_3_O_4_/KH560/M1000.

EDS analysis was utilized to analyze the elements present in the samples. Figure 4a–c illustrated the elemental distributions of Fe_3_O_4_(0.5 g)/KH560/M2070, Fe_3_O_4_(0.25 g)/KH560/M2070, and Fe_3_O_4_/KH560/M1000. The results demonstrated that the presence of C, N, and Si in Fe_3_O_4_ nanofluids was associated with the canopy polyether amines M2070/M1000 and the corona KH560. The results of EDS showed that the organic layer was successfully grafted on the surface of nanoparticles in all nanofluid systems.

**Figure 5:**
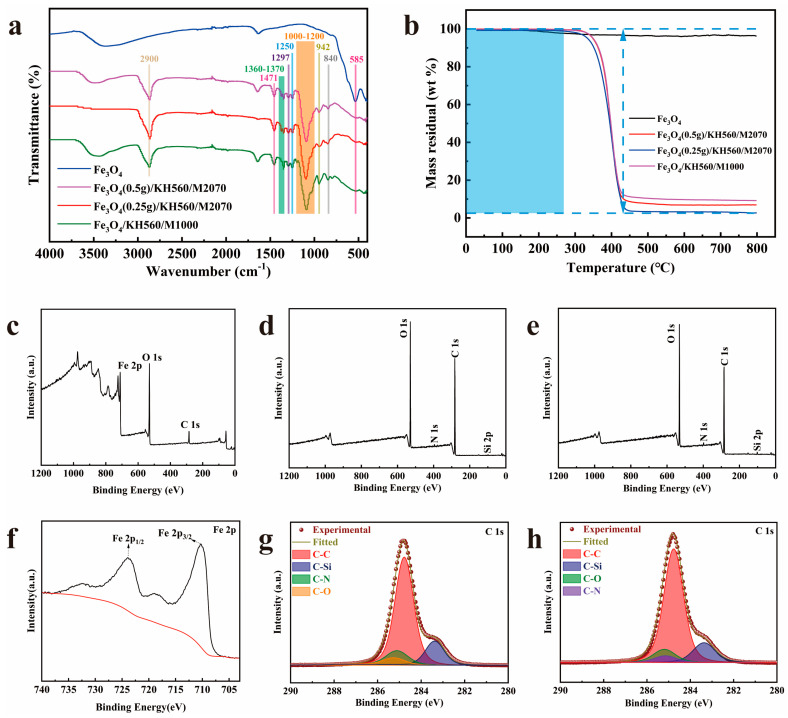
(**a**) FTIR spectra of Fe_3_O_4_, Fe_3_O_4_/KH560/M2070 and Fe_3_O_4_/KH560/M1000, (**b**) TGA curves of Fe_3_O_4_, Fe_3_O_4_/KH560/M2070 and Fe_3_O_4_/KH560/M1000. The wide scan XPS spectrum of (**c**) Fe_3_O_4_, (**d**) Fe_3_O_4_/KH560/M2070, and (**e**) Fe_3_O_4_/KH560/M1000. The narrow scan XPS spectrum of (**f**) Fe2p of Fe_3_O_4_, (**g**) C1s of Fe_3_O_4_/KH560/M2070, (**h**) C1s of Fe_3_O_4_/KH560/M1000.

Figure 5a shows the FTIR spectra of Fe_3_O_4_ powder nanoparticles, Fe_3_O_4_(0.25 g)/KH560/M2070, Fe_3_O_4_(0.5 g)/KH560/M2070, and Fe_3_O_4_/KH560/M1000. The peak at 585 cm^−1^, which is obviously present in Fe_3_O_4_ powder nanoparticles, was attributed to the stretching vibrations of Fe-O of Fe_3_O_4_ nanoparticles. All nanofluids have the same Fe_3_O_4_ core and KH560 corona, the difference lies in the canopy of polyetheramines M2070 and M1000. The overall structures of these two polyetheramines are relatively similar, the difference lies in the length of molecular chains, so the characteristic peaks of these two nanofluids are also similar. Taking the FTIR spectrogram of the nanofluid with the canopy of M2070 as an example, it can be seen that there are obvious characteristic peaks in the figure. The stretching and in-plane bending vibrations of C–H corresponded to the characteristic peaks at 2900 cm^−1^ and 1471 cm^−1^. The vibration of –CH_3_ that belongs to M2070 was associated with the double peaks at 1360 cm^−1^ and 1370 cm^−1^. Moreover, the vibrations of C–O–C and Si–O–Si (C) were related to the observed peaks at 1297 cm^−1^ and 1000–1200 cm^−1^, respectively. The presence of peaks at 942 cm^−1^ and 840 cm^−1^ was from Si–OH bands and O–Si–O. Most importantly, the characteristic peak at 1250 cm^−1^ belonged to the secondary amine group, which proved that the primary amine groups of M2070 and M1000 reacted with the epoxy groups of KH560. Therefore, comparing these curves, there were some peaks in nanofluids curves that were only related to KH560 and M2070. The appearance of the above characteristic peaks proved that the organic layer had been successfully grafted onto the surface of nanoparticles. The findings were consistent with those of SEM images.

In order to observe the thermal stability of Fe_3_O_4_ and magnetic solvent-free nanofluid, the samples were tested for thermal weight loss performance and TGA curves were made, as shown in Figure 5b. As shown in Figure 5b, the mass loss from 230 °C of Fe_3_O_4_ nanoparticles was due to the transition of Fe_3_O_4_ to γ-Fe_3_O_4_ [28]. Furthermore, in Fe_3_O_4_ nanofluids TGA curves from Figure 5b, there was almost no weight loss below 300 °C. This indicated that the Fe_3_O_4_ nanofluids were in a solvent-free, liquid-like state at room temperature. It also suggested that the Fe_3_O_4_ was protected by the organic layer and did not transform as the temperature increased. In the range of 350 °C to 450 °C, the weight of the Fe_3_O_4_ nanofluids was significantly decreased, due to the decomposition of organic layers in Fe_3_O_4_ nanofluids. After about 600 °C, the weight of the Fe_3_O_4_ nanofluids was no longer affected by temperature, indicating that the decomposition of the organic chains grafted on the surface of Fe_3_O_4_ had been completed and only the core Fe_3_O_4_ remained.

The XPS spectra of Fe_3_O_4_ nanoparticles, Fe_3_O_4_(0.5 g)/KH560/M2070, and Fe_3_O_4_/KH560/M1000 are presented in Figure 5c–h. Compared with the wide-scan spectrum of Fe_3_O_4_ nanoparticles in Figure 5c, Figure 5d,e referring to the wide scan XPS curves of Fe_3_O_4_ nanofluids, verifies the successful introduction of N and Si elements. To be specific, the observed peaks at 100 eV and 284 eV were associated with Si2p and C1s, respectively. The peaks at 398 eV and 532 eV were in relation to N1s and O1s. For the Fe2p narrow scan spectrum of Fe_3_O_4_ nanoparticles in Figure 5f, Fe2p3/2 and Fe2p1/2 corresponded to the peaks at 710 eV and 724 eV, which was reported by Hawn and Muhler [29,30]. Notably, in Figure 5d,e, the presence of the Fe peaks was obviously not detected in the wide scan spectra of Fe_3_O_4_ nanofluids, which suggested that the organic layers were grafted on the surface of nanoparticles. Moreover, through Gaussian curve fitting, the C1s spectra of Fe_3_O_4_ nanofluids were separated into four peaks as illustrated in Figure 5g,h. C-C, C-Si, C-N, and C-O were related to the peaks at 284.8 eV, 283.4 eV, 285.1 eV, and 285.2 eV, respectively, suggesting that Fe_3_O_4_ nanofluids have been successfully synthesized.

To investigate the effect of the graft density of organic chains on the adsorption properties of the absorbents, the graft density was calculated by Formula (3).

(3) $$n=\frac{\omega_{0}/M_{0}}{\omega_{c}/M_{c}}$$

where n: graft density, the number of moles of organic matter grafted on the surface of per mol Fe_3_O_4_. *M_c_*: the molar mass of the nanoparticle core. *M*_0_: the molar mass of the organic outer layer. *ω_c_*: the mass percentage of nanoparticle core. *ω*_0_: the mass percentage of the organic outer layer.

The graft densities of Fe_3_O_4_ nanofluids are shown in Table 1. It can be seen that the graft density of Fe_3_O_4_(0.25 g)/KH560/M2070 was higher than that of Fe_3_O_4_(0.5 g)/KH560/M2070, suggesting that more organic chains were coated on the surface of Fe_3_O_4_(0.25 g) core.

**Figure 6:**
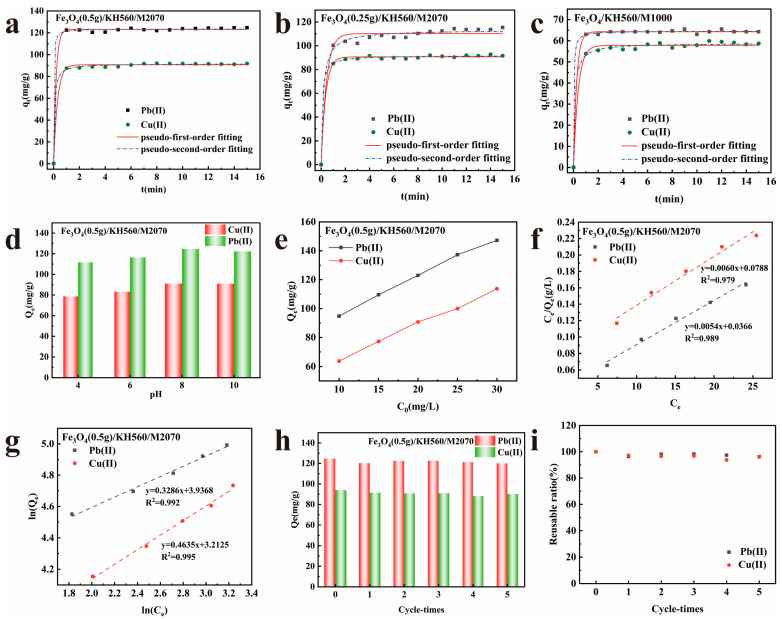
Adsorption capabilities and kinetic models of (**a**) Fe_3_O_4_(0.5 g)/KH560/M2070; (**b**) Fe_3_O_4_(0.25 g)/KH560/M2070; (**c**) Fe_3_O_4_/KH560/M1000 for Pb(II) and Cu(II) (pH = 8, C_0_ = 20 mg·L^−1^, T = 298 K); (**d**) Effect of pH on the adsorption capacity of Fe_3_O_4_(0.5 g)/KH560/M2070 on Cu(II) and Pb(II) (C_0_ = 20 mg·L^−1^, T = 298 K); (**e**) effects of initial concentration of Cu(II) and Pb(II) on the adsorption capacity of Fe_3_O_4_(0.5 g)/KH560/M2070 (pH = 8, T = 298 K); the (**f**) Langmuir and (**g**) Freundlich models of Fe_3_O_4_(0.5 g)/KH560/M2070 for Pb(II) and Cu(II) (pH = 8, C_0_ = 20 mg·L^−1^, T = 298 K); (**h**) the adsorption capacity of Fe_3_O_4_(0.5 g)/KH560/M2070 on Cu(II) and Pb(II) after 5 adsorption–desorption cycles (pH = 8, C_0_ = 20 mg·L^−1^, T = 298 K); (**i**) the reusable ratio of Fe_3_O_4_(0.5 g)/KH560/M2070 for Cu(II) and Pb(II) (pH = 8, C_0_ = 20 mg·L^−1^, T = 298 K).

To investigate the impact of grafting density and molecular chain length on the adsorption performance, the adsorption capabilities of Fe_3_O_4_(0.5 g)/KH560/M2070, Fe_3_O_4_(0.25 g)/KH560/M2070, and Fe_3_O_4_/KH560/M1000 for Pb(II) and Cu(II) were evaluated. As depicted in Figure 6a–c, Pb(II) and Cu(II) were rapidly adsorbed by the three adsorbents within the first 1 min, attributed to the abundance of active sites in the nanofluid and the excellent dispersibility of the solvent-free nanofluid in water with large contact area. However, after the initial 1 min, the adsorption capacities remained essentially unchanged, and the adsorption equilibrium was reached. This is attributed to the reduction in the number of active sites and the slowing of the adsorption rate as the reaction progresses, leading to minimal changes in adsorption capacity even with continued reaction. Additionally, the adsorption of Pb(II) by all three adsorbents was higher than the adsorption of Cu(II).

The difference between Fe_3_O_4_(0.5 g)/KH560/M2070 and Fe_3_O_4_(0.25 g)/KH560/M2070 lies in the grafting densities. As shown in Figure 6a,b, the two adsorbents exhibited similar adsorption capacities for Cu(II). However, the lower grafting density of Fe_3_O_4_(0.5 g)/KH560/M2070 resulted in better adsorption performance for Pb(II), with an equilibrium adsorption capacity of 124.68 mg·g^−1^. In contrast, the higher grafting density of Fe_3_O_4_(0.25 g)/KH560/M2070 led to lower equilibrium adsorption capacity for Pb(II) at 115.34 mg·g^−1^. This indicates that the two magnetic solvent-free nanofluids can effectively adsorb Pb(II) from water. Higher grafting density resulted in a higher amine content, leading to stronger chelation with heavy metals. However, due to the steric effect, the closer proximity of the groups led to spatial hindrance, resulting in poorer adsorption performance as grafting density increased.

The distinction between Fe_3_O_4_/KH560/M2070 and Fe_3_O_4_/KH560/M1000 lies in the molecular chain length. From Figure 6a,c, it is observed that the adsorbent with the canopy M2070 exhibited better adsorption performance. The nanofluid with the M2070 canopy achieved equilibrium adsorption capacity of 124.68 mg·g^−1^ for Pb(II), while the nanofluid with the M1000 canopy only achieved 65.48 mg·g^−1^ for Pb(II). This is because the organic molecular chain length of the adsorbent with the canopy of M2070 was longer, making the entire organic–inorganic system more flexible and increasing the free volume between the molecular chains, enabling better capture of heavy metal ions. Therefore, longer organic molecular chains result in better adsorption of Pb(II) and Cu(II) by the adsorbent.

The ratio of moles of bound heavy metal ions to amine groups in each of the adsorbents was calculated by Formula (4).

(4) $$n=\frac{Q_{t}M_{0}}{\omega_{0}M_{h}}$$

where *Q_t_*: the adsorption capacities at time t. *M*_0_: the molar mass of the organic outer layer. ω_0_: the mass percentage of the organic outer layer. *M_h_*: the molar mass of Cu(II) or Pb(II).

The ratio of moles of bound heavy metal ions to amine groups in each of the adsorbents is shown in Table 2. The results show that binding sites exceed the number of amine groups. Coordination may be occurring with the ether and alcohol oxygen atoms from the glycidyl moiety and/or physisorption. However, the ratio of moles of bound lead ions to amino groups in Fe_3_O_4_/KH560/M1000 was less than 1. This may be attributed to the shorter molecular chain of M1000, which resulted in relatively less free space between molecules during adsorption. Therefore, the reduced contact between lead ions and amino groups may result in some amino groups being unable to adsorb lead ions.

For better elucidation of the adsorption process of Fe_3_O_4_/KH560/M2070 on Pb(II) and Cu(II), the adsorption data were fitted by a pseudo-first-order (PFO) model and pseudo-second-order (PSO) model, which were expressed as follows [31]:

(5) $$Q_{t}=Q_{e}(1-e^{-K_{1}t})$$

(6) $$Q_{t}=\frac{K_{2}{Q_{e}}^{2}t}{1+K_{2}Q_{e}t}$$

where *Q_t_* (mg·g^−1^) and *Q_e_* (mg·g^−1^) were the adsorbed amount of Cu(II) and Pb(II) at t min and equilibrium. *K*_1_ (min^−1^) and *K*_2_ (g·mg^−1^·min^−1^) denoted the adsorption rate constants for the PFO and PSO models, respectively.

The fitting results were depicted in Figure 6a–c and the relevant kinetics parameters are summarized in Table 3. The correlation coefficients *R*^2^ were all greater than 0.992 as shown in the table, indicating excellent fits for both kinetic models. Notably, the R^2^ for PSO for all three adsorbents were higher than those for PFO. Compared to the PFO model, the PSO model demonstrated better fitting results. The results indicated that chemical adsorption predominates in the adsorption systems of the three adsorbents, likely due to the strong complexation and coordination interactions between the adsorbent amine groups and heavy metal ions [32].

The pH was a critical factor influencing the adsorption performance of adsorbents because of its influence on complexation, metal ion speciation, surface charge, and binding sites of the adsorbent. The relationship between the equilibrium adsorption quantity (*Q_e_*) of Fe_3_O_4_(0.5 g)/KH560/M2070 adsorbent on the heavy metal ions and pH was illustrated in Figure 6d. It was evident that the adsorption capacity of Fe_3_O_4_(0.5 g)/KH560/M2070 for heavy metal ions increases with the pH ranging from 4 to 8. At low pH, the adsorption capacity of Fe_3_O_4_(0.5 g)/KH560/M2070 was low due to the protonation of the amine groups to –NH^2+^– under low pH conditions and competition between H^+^ and metal cations for the active sites of the adsorbent surface [33,34].

As the pH increases, the adsorption capacity of the adsorbent improves due to the reduction of H^+^, enhancing the interaction between adsorption sites and metal ions. When the pH reaches 8, the deprotonation of the adsorbent surface results in a negative surface charge, facilitating electrostatic interactions with metal cations. Therefore, the maximum adsorption capacity of Fe_3_O_4_(0.5 g)/KH560/M2070 for Cu(II) and Pb(II) appeared at pH = 8. However, the adsorption capacity decreased at pH = 10, as a result of metal precipitation. Based on the above analysis, experiment was conducted at pH = 8.

As can be seen in Figure 6e, the adsorption capacity of Fe_3_O_4_(0.5 g)/KH560/M2070 for Cu(II) and Pb(II) improved as the concentration of the heavy metal ions increased from 10 to 30 mg·L^−1^. This was because the mass transfer of driving force increased at a higher initial concentration of metal cations in the solution [35].

Langmuir and Freundlich models were applied to explore nature of the adsorbent interacting with metal ions. The two models of Langmuir and Freundlich isotherm were generally expressed by Formulas (7) and (8) as follow [36,37].

(7) $$\frac{1}{Q_{e}}=\frac{1}{Q_{m}}+\frac{1}{K_{L}Q_{m}C_{e}}$$

(8) $$LnQ_{e}=LnK_{F}+\frac{1}{n}LnC_{e}$$

where *Q_e_* (mg·g^−1^) and *Q_m_* (mg·g^−1^) presented equilibrium adsorption capacity and the saturated adsorption capacity, respectively. *C_e_* (mg·L^−1^) was the equilibrium concentration after adsorption. *K_L_* (L·mg^−1^) and *K_F_* (L·mg^−1^) stood as the Langmuir and Freundlich model constant, respectively. n was the constant of heterogeneity.

For Langmuir model, the linear plot feature of *C_e_*/*Q_e_* versus *C_e_* was obtained in Figure 6f. For Freundlich model, the linear plot feature of ln*C_e_* versus ln*Q_e_* was achieved in Figure 6g. The calculated parameters of the two isotherm models were presented in Table 4. Actually, the adsorption process of Fe_3_O_4_(0.5 g)/KH560/M2070 tended to be heterogeneous multilayers adsorption, due to a slight advantage in R^2^ of the Freundlich adsorption isotherm. Both values of 1/*n* of Freundlich isotherms were less than 1 in Table 4, implying that the adsorption of Fe_3_O_4_(0.5 g)/KH560/M2070 on Cu(II) and Pb(II) easily proceed. Furthermore, the saturated adsorption capacity *Q_m_* of Fe_3_O_4_(0.5 g)/KH560/M2070 reached 166.67 and 185.19 mg·g^−1^ for Cu(II) and Pb(II), respectively, which was superior to most previously reported adsorbents, as demonstrated in Table 5.

The reusability of adsorbents holds significant importance in practical applications. After five adsorption–desorption cycles, the adsorption performance of Fe_3_O_4_/KH560/M2070 on Cu(II) and Pb(II) was depicted in Figure 6h. It was evident from Figure 6h that after the first cycle, the adsorption performance of Fe_3_O_4_/KH560/M2070 decreased for both Cu(II) and Pb(II). This phenomenon can be attributed to the strong coordination interaction between the amine groups of Fe_3_O_4_/KH560/M2070 and the metal ions, leading to permanent inactivation of some adsorption sites and incomplete desorption. Additionally, the loss of adsorbent may also contribute to the reduction in adsorption capacity. Furthermore, the reusable ratio (%) was calculated according to Equation (9).

(9) $$reusable\;ratio=Q_{n}/Q_{0}\times 100$$

where *Q*_0_ (mg·g^−1^) and *Q_n_* (mg·g^−1^) represented the equilibrium adsorption capacity at the initial time and after n adsorption–desorption cycles. After five cycles of adsorption–desorption, the reusable ratio of Fe_3_O_4_/KH560/M2070 for Cu(II) and Pb(II) was presented in Figure 6i. It can be observed that even after five cycles of adsorption–desorption, the reusable ratio of Fe_3_O_4_/KH560/M2070 for Cu(II) and Pb(II) remained more than 93%. Therefore, the adsorption–desorption experiments demonstrated the outstanding stability and reusability of Fe_3_O_4_/KH560/M2070, indicating its potential attractiveness in practical applications.

**Figure 7:**
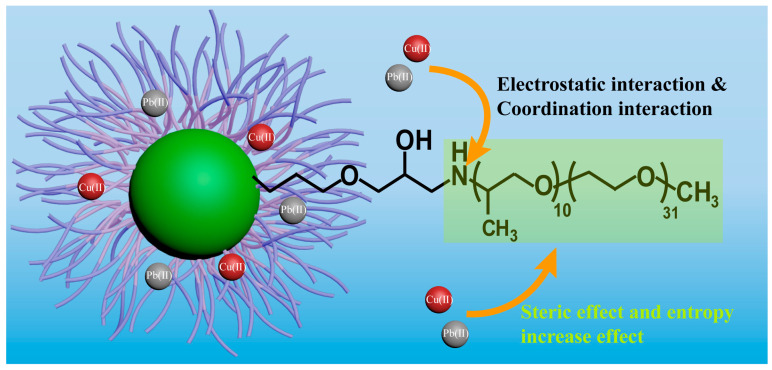
The illustration of the adsorption mechanism of the adsorbents for Cu(II) and Pb(II).

The adsorption mechanisms of the adsorbents for Cu(II) and Pb(II) can be summarized as follows, as depicted in Figure 7. Due to the grafted flexible organic chain, the dispersibility of the adsorbent was improved and more adsorption sites were provided for heavy metal adsorption. The nitrogen atom in the amine functional groups on the adsorbent form coordination interactions with the heavy metal ions, leading to the formation of relatively stable coordination bonds. Therefore, the presence of nitrogen atoms on the nano-adsorbent increased its adsorption capacity. Furthermore, at high pH, deprotonation of the adsorbent surface made the adsorbent surface negatively charged and facilitated electrostatic interactions with metal cations. Additionally, the enhancement of the adsorption performance was attributed to steric effect; specifically, the free volume of the outer organic chains increased due to the lower grafting density of Fe_3_O_4_(0.5 g)/KH560/M2070, which improved the opportunity for the metal cations to contact with the adsorbents, thereby increasing the adsorption properties. Moreover, the linking of oligomers to Fe_3_O_4_ induces a specific structural arrangement of these chains, thereby reducing entropy and favoring the adsorption process.

## 4. Conclusions

In this study, the synthesis of three solvent-free magnetic nanofluid adsorbents, Fe_3_O_4_(0.5 g)/KH560/M2070, Fe_3_O_4_(0.25 g)/KH560/M2070, and Fe_3_O_4_/KH560/M1000, was successfully achieved. The use of (3-glycidoxypropyl) trimethoxysilane (KH560) and polyetheramine M2070 as flexible organic layers endowed the adsorbents with flow properties at room temperature. Characterization of the adsorbents’ morphology, structure, and properties was carried out using SEM, TEM, EDS, FTIR, XPS, and TGA analyses. Furthermore, the adsorption properties of the adsorbents towards Cu(II) and Pb(II) in aqueous solutions were investigated. Notably, Fe_3_O_4_(0.5 g)/KH560/M2070 exhibited adsorption capacities of 92.02 mg·g^−1^ for Cu(II) and 124.68 mg·g^−1^ for Pb(II). Additionally, adsorption kinetics were studied, with the PSO model providing a better fit to the experimental data. The results indicated that the adsorption of heavy metal ions by the adsorbent was primarily attributed to chemical adsorption. Furthermore, the magnetic loading conferred excellent recyclability to the adsorbent. Multiple adsorption mechanisms, including coordination interactions, electrostatic interaction, steric effect, and entropy were found to simultaneously contribute to the adsorption process.

## Figures and Tables

**Table 1:** The graft density of the organic layer of the adsorbents.

Samples	ω_c_ (%)	ω_0_ (%)	M_c_	M_0_	n
Fe_3_O_4_(0.5 g)/KH560/M2070	6.9	93.1	232	2236	1.40
Fe_3_O_4_(0.25 g)/KH560/M2070	3.2	96.8	232	2236	3.14
Fe_3_O_4_/KH560/M1000	9.7	90.3	232	1236	1.75

**Table 2:** The ratio of moles of bound heavy metal ions to amine groups in each of the adsorbents.

Samples	Cu(II)	Pb(II)
Fe_3_O_4_(0.5 g)/KH560/M2070	3.45	1.45
Fe_3_O_4_(0.25 g)/KH560/M2070	3.35	1.29
Fe_3_O_4_/KH560/M1000	1.28	0.43

**Table 3:** Kinetic parameters for adsorption of Cu(II) and Pb(II) by adsorbents.

Adsorbents	Models	Parameters	Cu(II)	Pb(II)
Fe_3_O_4_(0.5 g)/KH560/M2070	PFO	*Q_e_* (mg g^−1^)	90.70	123.11
		*K*_1_ (min^−1^)	3.297	5.191
		R^2^	0.997	0.998
	PSO	*Q_e_* (mg g^−1^)	91.70	123.56
		*K*_2_ (g mg^−1^ min^−1^)	0.175	0.438
		*R* ^2^	0.998	0.999
Fe_3_O_4_(0.25 g)/KH560/M2070	PFO	*Q_e_* (mg g^−1^)	90.66	110.24
		*K*_1_ (min^−1^)	2.748	2.279
		R^2^	0.997	0.981
	PSO	*Q_e_* (mg g^−1^)	91.80	113.39
		*K*_2_ (g mg^−1^ min^−1^)	0.137	0.052
		*R* ^2^	0.998	0.992
Fe_3_O_4_/KH560/M1000	PFO	*Q_e_* (mg g^−1^)	57.81	64.25
		*K*_1_ (min^−1^)	2.606	3.898
		*R* ^2^	0.992	0.998
	PSO	*Q_e_* (mg g^−1^)	58.83	64.56
		*K*_2_ (g mg^−1^ min^−1^)	0.158	0.553
		*R* ^2^	0.996	0.999

**Table 4:** Parameters for isothermal model of Cu(II) and Pb(II) adsorption by Fe_3_O_4_(0.5 g)/KH560/M2070.

Adsorbate	Langmuir			Freundlich		
	*Q_m_* (mg·g^−1^)	*K_L_* (L·mg^−1^)	*R* ^2^	*K_F_* (L·mg^−1^)	*n*	*R* ^2^
Cu(II)	166.67	0.076	0.979	24.8	2.157	0.995
Pb(II)	185.19	0.148	0.989	51.3	3.043	0.992

**Table 5:** Comparison of adsorption capacity of Fe_3_O_4_(0.5 g)/KH560/M2070 with other adsorbents.

Adsorbents	*Q_m_* (mg·g^−1^)/Cu(II)	*Q_m_* (mg·g^−1^)/Pb(II)	Ref.
CTPC	81.97	123.46	[38]
Fe_3_O_4_@PDA	86.35	57.25	[39]
SBA-NPA	48.26	106.62	[40]
CB-G_3_	88.82	97.87	[41]
SiO_2_-NH-HPBT	20.5	68.6	[42]
P-PAN fibers	131.41	176.99	[43]
Fe_3_O_4_(0.5 g)/KH560/M2070	166.67	185.19	This work

## Data Availability

The original data are available from the corresponding authors upon reasonable request.
